# Guardians of the light: The redox regulation of the PSI during photosynthesis

**DOI:** 10.1093/plphys/kiae482

**Published:** 2024-09-12

**Authors:** Sara Selma

**Affiliations:** Assistant Features Editor, Plant Physiology, American Society of Plant Biologists; VIB Center for Plant Systems Biology, 9052 Ghent, Belgium

As photosynthetic organisms, plants must optimize their molecular strategies to adapt to the ever-changing light conditions in their environment. An interplay of 3 alternative electron-transport pathways—linear, cyclic, and pseudocyclic (Mehler reaction)—can occur in the cell to maximize the flexibility of photosynthesis against light photoperiod (Allen 2003). The pseudocyclic electron flow photoreduces O_2_ and generates of reactive oxygen species (ROS) in PSI and is predominant in the short-day photoperiod (Michelet and Krieger-Liszkay 2012). ROS can play a role as signal molecules but also can be potentially harmful (Foyer and Hanke 2022). To manage light-induced ROS and maintain proper photosynthetic function, thiol-dependent redox enzymes play a crucial role in chloroplast redox regulation in the PSI (Buchanan 2017). It has been shown that key redox enzymes involved in the control of the levels of ROS generated during photosynthesis are the m-type thioredoxins (Trx), NADPH-dependent reductase C (NTRC), and 2-Cys peroxiredoxin (2-Cys PRX). The interaction of these enzymes functions as a redox regulatory network, where 2-Cys PRX can be reduced by NTRC and Trx but can also re-oxidize the reduced Trx, providing a rapid acclimation system to variations in the photosynthetic electron flow due to changes in the light regime (Telman et al. 2020). Proteins regulating the redox state of the thylakoid lumen include the Lumen Thiol Oxidoreductase 1 (LTO1), a transmembrane protein with a Trx-like domain, and the atypical cytochrome c6A (Marcaida et al. 2006). However, it is unclear how the redox state of the stroma is transmitted to the thylakoid lumen or which players are involved in the redox regulation of PSI.

In this issue of *Plant Physiology*, Hani et al. present a new model of the redox regulation of PSI during photosynthesis in different photoperiods. The authors highlight the role of the proteins NTRC, Trx, and 2-Cys PRX in controlling the redox state of the protein PsaF and the O_2_-reduction capacity of PSI, thus regulating ROS generation during photosynthesis.

As a starting point, the authors compared the ROS production generated in the light in *Arabidopsis thaliana* (Arabidopsis) leaves and isolated thylakoid grown under short-day (SD, 8 h light, 16 h dark) and long-day (LD, 16 h light, 8 h dark) conditions employing spin-trapping assay that detected hydroxyl radicals derived from superoxide anion radicals and hydrogen peroxide. The amount of ROS generated under SD conditions was about twice that of LD conditions, due to additional electron transport in SD plants that generates a higher proton gradient and more ATP compared with LD plants (Michelet and Krieger-Liszkay 2012). A previous work also showed that this difference in ROS generation is not observed when the thylakoids grown in SD and LD were treated with an uncoupler, a molecule that disrupts the photophosphorylation in chloroplasts by dissociating the reactions of ATP synthesis from the electron transport chain, increasing the ROS regeneration in LD thylakoids but maintaining the ROS levels in SD conditions (Lepistö et al. 2009). These results support the differential regulation of the PSI during SD, enhancing the O_2_ reduction due to the electron transport chain and thus maintaining the pH gradient and ATP systhesis through the action of the pseudocyclic electron flow.

After confirming the differential ROS production between SD and LD conditions, the authors explore the effect of NTRC, Trx m, and 2-Cys PRX proteins in regulating ROS levels. Loss-of-function Arabidopsis mutants in Trx m isoforms (*trxm4*and the double mutant *trxm1m2*) or NTRC showed that the difference in ROS production between SD and LD was lost. In the case of Trx m mutants, the O_2_ reduction was low, generating ROS production similar of LD plants. On the other hand, the NTRC mutants showed high O_2_ reduction, generating high amounts of ROS like the SD plants.

Also, the overexpression of NTRC enhanced the difference in ROS levels between SD and LD. However, although mutants lacking 2-Cys PRX showed an increase in ROS, the difference between SD and LD remained. Further investigation revealed that the localization of the studied proteins changes between SD and LD. In the case of 2-Cys PRX, the authors found that the presence of the protein in the thylakoid membrane is strongly determined by the photoperiod. LD-grown plants showed a slightly more abundant amount of 2-Cys PRX but also bound to the thylakoid membrane in its dimeric (oxidized) form. However, in the PSI preparations, the 2-Cys PRX was not found by inmuno-detection, suggesting that this protein is not directly associated to the PSI complexes.

In contrast, Trx m4 and Trx m2 were found attached to the thylakoid membranes in SD but not LD conditions, which could explain their efficient electron transport to O_2_ at PSI in SD. Based on these results, the authors hypothesized that the key to understanding the differential regulation of PSI by the Trx-NTRC complex could be a thiol-dependent redox enzyme present in PSI itself. Redox western assays were performed to reveal the oxidative state of the candidate members of PSI, showing that the protein PsaF, a transmembrane protein that contains at its luminal site 2 cysteine residues, can change its redox state. The relative abundance of the 2 redox species of PsaF can be found differentially between dark and light, suggesting that the change in the redox state of this protein drives the day-length regulation of the electron transport in PSI. Finally, the authors pointed to the involvement of transmembrane proteins CCDA and HCF164 in transmitting the redox state from the stroma to the thylakoid lumen thiol groups of PsaF. This was shown with the Arabidopsis mutants of CCDA and LTO1, which lost the SD–LD difference in reduced O_2_ production.

In summary, Hani et al. highlight the significant impact of photoperiod on ROS production, identifying the thiol-dependent enzymes involved in the pseudocyclic electron transport chain. The novel findings of this study allow the generation of a new model of redox regulation of the PSI (Fig.). This model can guide further research to investigate the redox regulation of the alternative electron-transport pathways under fluctuating light conditions or abiotic stress.

**Figure.**
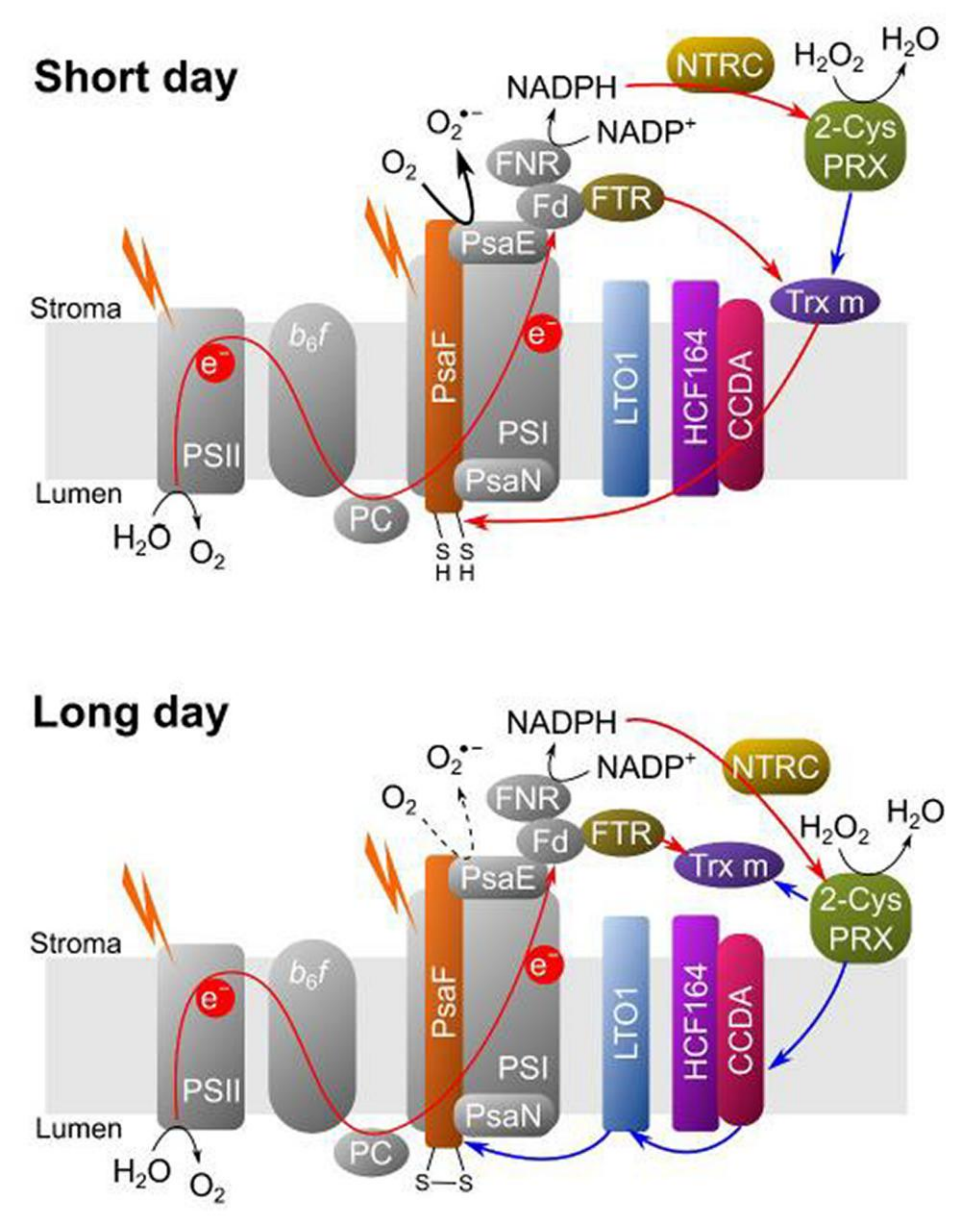
Model of redox regulation of O_2_ reduction at PSI. Reduced cysteine residues in PsaF favor higher O_2_ reduction activity. The redox state of PsaF is regulated by 2 thiol-modulating redox systems: one responsible for reducing the disulfide bond and the other for oxidizing it. Trx m plays a central role in controlling the reduction state of PsaF, and Trx m itself is reduced by FTR. Under SD conditions, Trx m associates with the thylakoid membrane and reduces CCDA, which reduces via HCF164 and finally PsaF. In LD conditions, reduced PsaF is oxidized by LTO1, which is oxidized by CCDA (blue arrows); 2-Cys-PRX attaches to the membrane, allowing the oxidation of CCDA. In addition, it can oxidize Trx m (blue arrows). Image extracted from Hani et al. 2024.
